# Cardiovascular disease and hypertension in sub-Saharan Africa: burden, risk and interventions

**DOI:** 10.1007/s11739-016-1423-9

**Published:** 2016-03-21

**Authors:** Francesco Paolo Cappuccio, Michelle Avril Miller

**Affiliations:** Division of Health Sciences (Mental Health and Wellbeing), WHO Collaborating Centre, University of Warwick, Gibbet Hill Road, Coventry, CV4 7AL UK

**Keywords:** Sub-Saharan Africa, Cardiovascular disease, Hypertension, Salt reduction, Drug therapy

## Abstract

Cardiovascular disease, including stroke, heart failure and kidney disease, has been common in sub-Saharan Africa for many years, and rapid urbanization is causing an upsurge of ischaemic heart disease and metabolic disorders. At least two-thirds of cardiovascular deaths now occur in low- and middle-income countries, bringing a double burden of disease to poor and developing world economies. High blood pressure (or hypertension) is by far the commonest underlying risk factor for cardiovascular disease. Its prevention, detection, treatment and control in sub-Saharan Africa are haphazard and suboptimal. This is due to a combination of lack of resources and health-care systems, non-existent effective preventive strategies at a population level, lack of sustainable drug therapy, and barriers to complete compliance with prescribed medications. The economic impact for loss of productive years of life and the need to divert scarce resources to tertiary care are substantial.

## Introduction

Despite significant improvement in health research in the African region since 2000 [1], the quality of information on the determinants of health and disease and of studies of implementation of effective preventive and therapeutic strategies remain scanty. The Global Burden of Disease, Injuries and Risk Factor Study is the first systematic and comprehensive attempt to map and quantify risk factors and diseases to identify emerging threats to population health and opportunities for prevention [2–5]. Of particular interest, the analyses of the burden of death and disability attributable to modifiable risk factors have identified emerging threats in risk factors traditionally seen in developed countries, like tobacco smoking, obesity and high salt intake [2–7]. They explain the surge in the burden of cardiovascular disease (CVD) in sub-Saharan Africa, namely hypertension, renal disease, and heart failure. This upsurge of the CVD epidemic poses an additional burden on the already over-burdened health-care systems in these settings creating critical challenges to both national health systems and policy development that can impede the development of a strategic plan to address the CVD epidemic.

## The burden of cardiovascular disease in sub-Saharan Africa

**Fig. 1:**
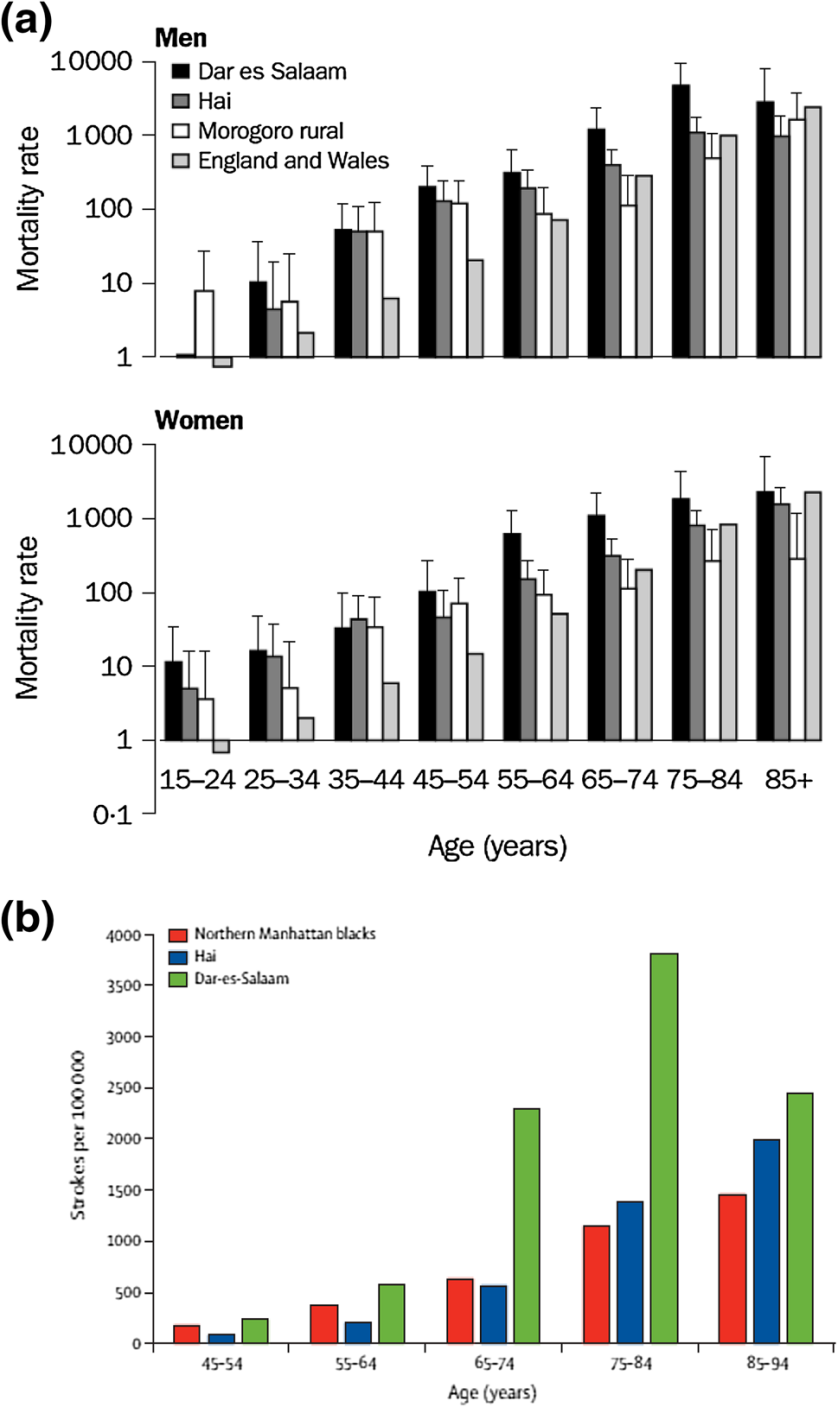
**a** Number and yearly rate (per 100,000) of deaths from stroke in 10-year age-bands in adult men and women in Dar-es-Salaam, Hai district, Morogoro rural district, and England and Wales (1992) (from Walker et al. [13]). **b** Age-specific stroke rates for people aged 45 years or over in Hai, Dar-es-Salaam, and black people in Northern Manhattan (from Walker et al. [14])

CVD is a major global public health crisis, being responsible for 30 % of worldwide deaths in 2008 (17 million deaths worldwide from an annual total of 57 million deaths) with an alarming 80 % of these deaths occurring in low- and middle-income countries (LMICs) [8]. Whilst effective measures are being put in place in high-income countries resulting in a decline in the rate of CVD [9], CVD mortality is on a steady rise in LMICs with rates of up to 300–600 deaths attributed to CVD per 100,000 population, and is projected to increase causing preventable loss of lives [8]. The uncontrolled CVD epidemic is associated with increasing socio-economic costs with high levels of disability and loss of productivity, exacerbating poverty and increasing health inequalities. The poor have the worst outcomes from CVD, largely because of their inability to access to or afford preventive services and ongoing treatments. Much of the population risk of CVD is attributable to nine modifiable traditional risk factors, including smoking, history of hypertension or diabetes, obesity, unhealthy diet, lack of physical activity, excessive alcohol consumption, raised blood lipids and psychosocial factors [10]. Eight of these risk factors (excessive alcohol use, tobacco use, high blood pressure, high body mass index (BMI), high cholesterol, high blood glucose, dietary choices and physical inactivity) account for 61 % of CVD deaths globally. About 84 % of the total global burden of disease they cause occurs in LMICs, with studies showing that alleviating exposure to these eight risk factors would improve global life expectancy by almost 5 years [11, 12]. In 2010, the three leading risk factors for global disease burden were high blood pressure [7.0 % of global disability-adjusted life-years (DALYs), where a DALY is to be considered as a year of healthy life lost], tobacco smoking including second-hand smoke (6.3 %), and household air pollution from solid fuels (4.3 %) [3]. Dietary risk factors and physical inactivity collectively accounted for 10.0 % of global DALYs in 2010, with the most prominent dietary risks being diets low in fruits and those high in sodium (or salt) [3]. Thus, further elucidation of the role of these risk factors is important for developing clear and effective strategies for improving global health. Notwithstanding the importance of communicable diseases, child and maternal health and malnutrition, unsafe water and sanitation, and malaria as leading causes of loss of DALYs, chronic disease has seen a rapid increase in sub-Saharan Africa from 1990 to 2010, ranking in the first few places [1]. For example, stroke mortality rates, measured in both urban and rural Tanzania by validated verbal autopsies, were higher than those of England and Wales [13] and of black people in Northern Manhattan [14] (Fig. 1), suggesting that untreated hypertension is an important factor. Sub-Saharan Africa is experiencing a double burden of disease that calls for a more integrated approach for the detection, prevention and management of CVD in LMICs.

## Hypertension in sub-Saharan Africa

CVD is the leading cause of death worldwide and hypertension is the leading associated risk factor [2, 3]. High blood pressure accounts for 9.4 million deaths, more than elevated BMI, fasting plasma glucose, and total cholesterol combined [3]. As of 2008, almost 1 billion people had uncontrolled hypertension worldwide [15]. The African region has the highest prevalence rate, 46 % of adults aged 25 and above. It has been suggested that the prevalence of CVD and hypertension are increasing rapidly in sub-Saharan Africa [16]. The current prevalence in many developing countries, particularly in urban areas, is already as high as that seen in developed countries [17, 18]. The number of adults with hypertension in 2025 is predicted to increase by about 60 % to a total of 1.56 billion [18], with disproportionate prevalence in developing countries including sub-Saharan Africa. In contrast with other CVD risks such as high BMI, the burden of hypertension is greater in lower income countries than higher income settings [19]. Multiple risk factors positively interact to exacerbate CVD risks. Hypertension, for example, combined with unhealthy diets and lack of physical activity (sodium and alcohol consumption, high BMI, and low physical activity), has a multiplicative negative effect on CVD mortality and DALYs [3]. A substantial part of the CVD risk of death and DALYs attributable to these factors is not only exacerbated by, but also mediated through high blood pressure [3]. In LMICs, overweight or obesity are associated with high blood pressure or with progression to hypertension [20, 21]. Hypertension is also a determinant of chronic kidney disease (CKD), a recognized marker of the poor health outcomes of hypertension and diabetes [22]. Moreover, growing evidence indicates that CKD is a strong cardiovascular risk factor in itself [23]. In sub-Saharan Africa cardiovascular and renal disease are important contributors to morbidity and mortality (up to a quarter), both among acute medical admissions and among outpatient hypertensives, in whom renal disease is an important complication [24].

**Fig. 2:**
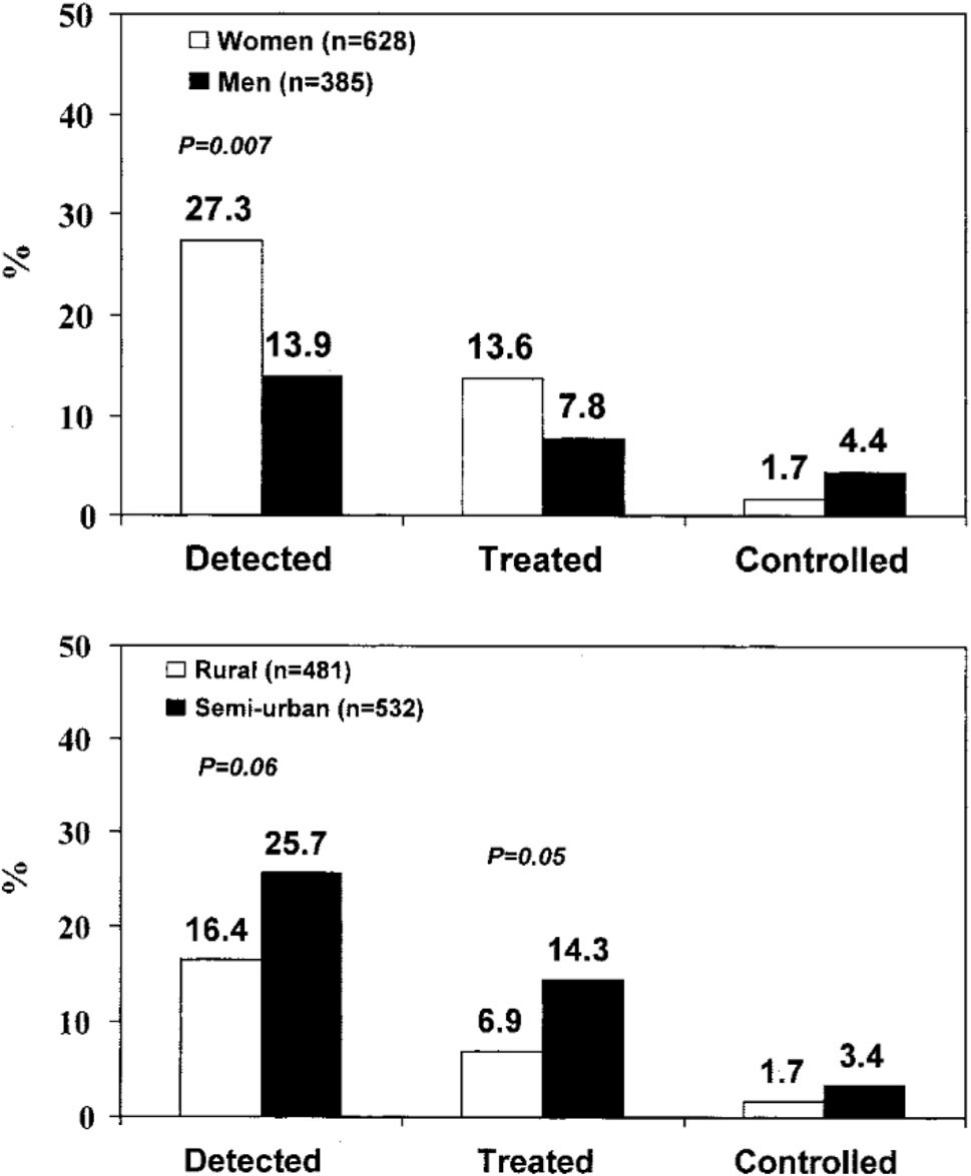
*Top* proportion of people with hypertension, who were detected, treated, and controlled (i.e., BP 140 and 90 mmHg) by gender and age group in Ashanti, West Africa. Hypertension is defined as systolic BP 140 and/or diastolic BP 90 mmHg, or on anti-hypertensive medication. *Bottom* proportion of people with hypertension, as defined above, who were detected, treated, and controlled (i.e., BP 140 and 90 mmHg) in rural and semi-urban villages by age group (from Cappuccio et al. [28])

In sub-Saharan Africa, the prevention, detection, management and control of hypertension should now be regarded as a high priority [25]. It is estimated that if the 10–20 million people who are believed to have hypertension in sub-Saharan Africa were treated effectively, about 250,000 deaths would be prevented annually [25]. Sadly, repeated reports over the years indicate that although the prevalence of hypertension has reached—and in some parts of Africa overcome—that seen in the developed world, the prevention, detection, management and control of high blood pressure are haphazard and insufficient [26, 27]. In a large community-based survey of adults in rural and semi-urban Ghana, the prevalence of hypertension is 28.7 % overall, comparable in men and women, but higher in semi-urban compared to rural villages (32.9 versus 24.1 %), and it increases with age [28]. Detection rate is low, lower in men than women (13.9 versus 27.3 %) (Fig. 2). Treatment and control rates are low in both groups (7.8 and 4.4 versus 13.6 and 1.7 %). Detection, treatment, and control rates are higher in semi-urban (25.7, 14.3, and 3.4 %) than in rural villages (16.4, 6.9, and 1.7 %). The data confirm that hypertension is common in adults in Ghana, particularly in areas of rapid urbanization. Detection rates are suboptimal in both men and women, especially in rural areas. Adequate treatment of high blood pressure is at a very low level [28].

## Reduction in salt consumption in sub-Saharan Africa

**Fig. 3:**
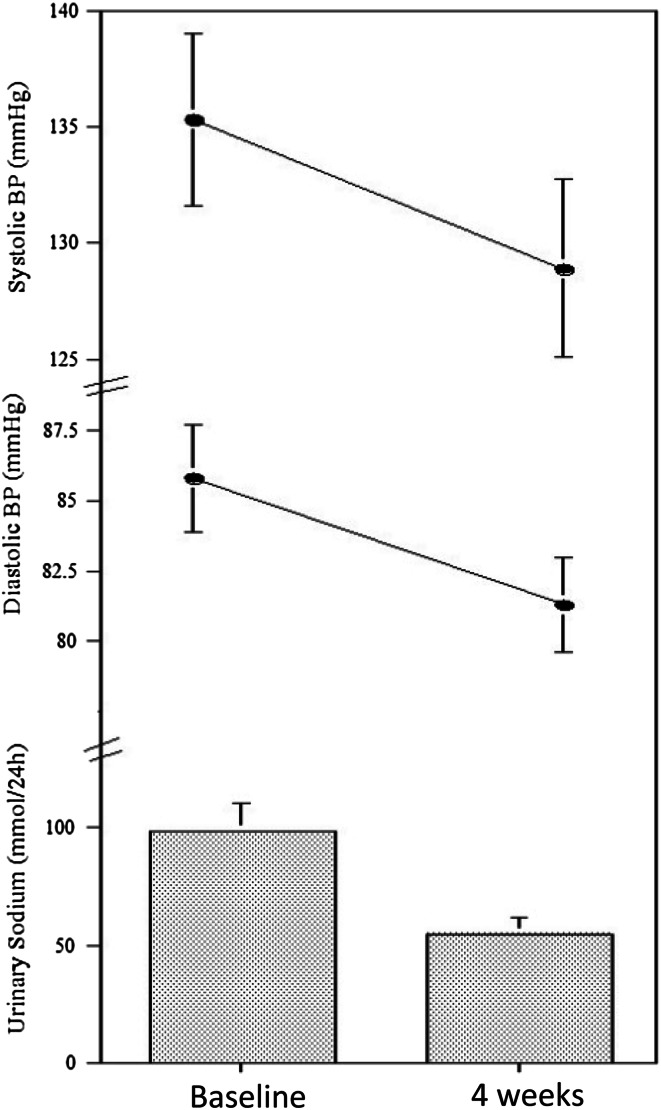
Effects of a 4-week intervention aiming at reducing dietary salt consumption in 20 adult farmers in the Ashanti region of Ghana (from Cappuccio et al. [33])

In Africa, the reduction in population attributable risk when blood pressure is lowered is 13 times greater than in the USA [25]. However, in places where there is poor health-care provision, the detection of hypertension is still haphazard and unreliable, and population-wide strategies to reduce blood pressure might have an important impact on the number of cardiovascular events—especially strokes, kidney disease and heart failure—in the community. There is good evidence that a reduction in salt intake reduces blood pressure [29], and that black people are more sensitive than white people to the beneficial effect of reducing salt intake [30]. The Global Burden of Disease Study on Salt [7] estimates that 1.65 million deaths from cardiovascular causes that occurred in 2010 are to be attributed to salt consumption above a reference level of 2.0 g of sodium (equivalent to 5 g of salt) per day, and that they could have been averted by a moderate population reduction in salt consumption. In the western world, notwithstanding this good evidence, it has been difficult to implement successful salt reduction strategies in the population, since most of the salt ingested is in processed food [30, 31]. So, any intervention would involve the participation of the food industry [31]. In contrast, in populations whose intake of processed food is negligible—such as in semi-urban and rural sub-Saharan Africa [32]—salt reduction strategies based on health promotion, increased awareness and behavioural changes in individuals and groups ought to be relatively easy to implement, and have a good chance of success [33]. Two trials in sub-Saharan Africa have confirmed that simple, cost-effective, and culturally adapted behavioural and educational interventions to reduce blood pressure with dietary salt reduction can be successfully implemented [33–35]. In the study in south west Nigeria 82 normotensive adults participants (49 men and 33 women) received dietary advice to reduce sodium intake and to maintain it for 2 weeks. Both blood pressure and 24 h urinary sodium excretion (a marker of intake) were measured before and after the intervention. Salt intake fell by approximately 4.6 g per day and systolic blood pressure by 4.7 mmHg in men and 7.0 mmHg in women [34]. In a similar pilot study in rural Ghana, 20 adult normotensive farmers (8 men and 12 women) participated in a salt reduction trial of 4-week duration [33]. Both blood pressure and 24 h urinary sodium excretion were measured at baseline and 4 weeks after attending a daily 1.5 h session of vigorous nutrition education for a week, followed by weekly sessions thereafter. At the end of the study there was a fall in salt consumption of about 50 % (approx. 2.9 g per day) despite the initial low salt consumption (average 5.8 g per day). At the same time blood pressure fell by 6.4/4.5 mmHg (Fig. 3). Finally, in a much larger and longer-term community-based cluster randomized trial of moderate reduction in salt consumption through health promotion in twelve rural and semi-urban villages in the Ashanti region of Ghana, over 1000 adult men and women took part in a 6-month intervention aiming at reducing the population blood pressure through health education on the detrimental effects of high salt consumption [35]. Blood pressure was measured at baseline, 3 and 6 months after the intervention took place. The intervention consisted of an intensive health education programme delivered by community health workers to villagers, daily for the first week of the study, and once weekly thereafter. The sessions were held in communal areas like churches, churchyards, schools and community centres. Salt intake was monitored by repeated 24 h urine collections throughout. After 6 months the intervention resulted in a fall in blood pressure of 2.5/3.9 mmHg. There was a fall in average population systolic blood pressure of 1.3 mmHg for just under 3 g of salt reduction. The impending epidemic of cardiovascular disease in sub-Saharan Africa in a context of lack of resources is a serious global public health challenge. Community-based strategies of health promotion for the management of chronic disease through lifestyle change in sub-Saharan Africa should be considered. Priority should be given to the cost-effective population salt reduction strategy, a global priority to reduce the burden of cardiovascular disease by 25 % by 2025 [30].

## Drug therapy in sub-Saharan Africa

There is sufficient evidence to suggest that the pathophysiology of hypertension varies by ethnic groups, so that the hypertension seen in people of African descent, whether living in Africa or migrated through the different diasporas, shows distinct features in terms of presentations, underlying mechanisms, response to treatments (pharmacological and not) and susceptibility to target organ damage and complications [36–38]. Hypertension in Africa is characterized, by and large, by low-renin levels [37]. The blunted response of the renin-angiotensin-aldosterone system to a variety of physiological stimulations makes the blood pressure response of people of African origin more sensitive to changes in salt intake (excessive raises for increases in salt intake and significant falls for reductions), and less responsive to drugs that reduce blood pressure by acting on inhibitions of pathways of the renin-angiotensin-aldosterone system [37]. In general, African patients do not respond as well as other groups to monotherapies with blockers of renin production (like beta-blockers) or drugs that block either the production (ACE-inhibitors) or the action (angiotensin-receptor blockers) of angiotensin II [39]. In ALLHAT, a randomized controlled clinical trial of different classes of anti-hypertensives used as monotherapy, black patients treated with ACE-inhibitors show the least blood pressure response and a blunted benefit on stroke incidence [40]. These shortcomings, however, are overcome once hypertension is treated in combination therapy [37]. So, for the management of hypertension in Africa, initiation treatment regimens based on diuretics and calcium-channel blockers should be the first choice.

Compliance with treatment is a particular issue for patients with chronic disease and for clinical staff, and more so in LMICs with scarce health-care facilities. Since it is difficult to sustain motivation for treatment, especially for asymptomatic conditions, how can patients with chronic conditions be helped to comply with treatment? Methods include identifying side effects, educating patients in the need for long-term management, simplifying treatment and involving the patient in agreeing to an acceptable drug regimen: these strategies are shown to improve compliance. Obviously, blood pressure control requires compliance with prescribed medications.

In sub-Saharan Africa the unaffordable cost of sustaining chronic management is the commonest cause of non-compliance with prescribed regimes and lack of blood pressure control [41]. However, forgetfulness and prejudice of untoward effects also play a role [42]. In addition to lack of affordability, likely predictors of non-compliance are level of educational attainment, and the tendency to seek alternative treatment through traditional medicine [42], suggesting a sceptical approach to the benefits of the conventional health-care provided by hospitals and clinics. The only available method of measuring compliance at clinics in resource-poor areas is patient-reported treatment taking, although this may result in over-estimation. Reporting by patient is a low-technology approach that reinforces health education by emphasizing the contribution of patients and health-care professionals in establishing treatment.

## Conclusions

In 2005 global health funding per death for HIV/AIDS was $1029 compared with $320 for non-communicable diseases [43], indicating that there is a widespread apathy with major health development funds, placing less emphasis in tackling non-communicable diseases in LMICs compared to other diseases. Thus, concerted global, regional and local partnerships are pivotal to address this silent epidemic. The United Nations General Assembly convened a high-level meeting on non-communicable diseases in New York in September 2011 to take action against this global epidemic [30]. As a result the World Health Organization was tasked with delivering a compelling agenda, now enshrined in the WHO Global Action Plan for the prevention and control of non-communicable diseases covering the period 2013–2020. National governments, policy-makers and international development partners have a key role in ensuring that CVD prevention and control become a major part of the health-care development agenda.

CVD creates an enormous impact on socio-economic development due to societal and global determinants [43, 44] as many of those in the high-risk group are at the peak of their productive and economic activity [45, 46]. These determinants include rapid globalization, unplanned urbanization, global trade and agricultural policies amongst other things, which ultimately influence an individual’s or a society’s ability to make healthy choices contributing to its negative impact on social and economic growth in LMICs [47]. The economic impact for loss of productive years of life and the need to divert scarce resources to tertiary care are substantial.

## References

[1] Uthman OA, Wiysonge CS, Ota MO (2015). Increasing the value of health research in the WHO African Region beyond 2015— reflecting on the past, celebrating the present and building the future: a bibliometric analysis. BMJ Open.

[2] Lozano R, Naghavi M, Foreman K (2012). Global and regional mortality from 235 causes of death for 20 age groups in 1990 and 2010: a systematic analysis for the Global Burden of Disease Study 2010. Lancet.

[3] Lim SS, Vos T, Flaxman AD (2012). A comparative risk assessment of burden of disease and injury attributable to 67 risk factors and risk factor clusters in 21 regions, 1990–2010: a systematic analysis for the Global Burden of Disease Study 2010. Lancet.

[4] Global Burden of Disease Study 2013 Collaborators (2015). Global, regional, and national incidence, prevalence, and years lived with disability for 301 acute and chronic diseases and injuries in 188 countries, 1990–2013: a systematic analysis for the Global Burden of Disease Study 2013. Lancet.

[5] GBD 2013 Risk Factors Collaborators (2015). Global, regional, and national comparative risk assessment of 79 behavioural, environmental and occupational, and metabolic risks or clusters of risks in 188 countries, 1990–2013: a systematic analysis for the Global Burden of Disease Study 2013. Lancet.

[6] Powles J, Fahimi S, Micha R, Global Burden of Diseases Nutrition and Chronic Diseases Expert Group (NutriCoDE) (2013). Global, regional and national sodium intakes in 1990 and 2010: a systematic analysis of 24 h urinary sodium excretion and dietary surveys worldwide. BMJ Open.

[7] Mozaffarian D J, Fahimi S S, Singh GM, Global Burden of Diseases Nutrition and Chronic Diseases Expert Group (NUTRICODE) (2014). Global sodium consumption and death from cardiovascular causes. N Engl J Med.

[8] World Health Organization (2011). Global status report on non-communicable diseases 2010.

[9] Ebrahim S, Pearce N, Smeeth L (2013). Tackling non-communicable diseases in low-and-middle-income countries: is the evidence from high-income countries all we need?. PLoS Med.

[10] Gersh BJ, Sliwa K, Mayosi BM, Yusuf S (2010). Novel therapeutic concepts: the epidemic of cardiovascular disease in the developing world: global implications. Eur Heart J.

[11] World Health Organization (2005). The role of CVD risk factors (WHO Global InfoBase Team). The SuRF report 2. Surveillance of chronic disease risk factors: country-level data and comparable estimates.

[12] World Health Organization (2009). Global health risks: mortality and burden of disease attributable to selected major risks.

[13] Walker RW, McLarty DG, Kitange HM, Adult Morbidity and Mortality Project (2000). Stroke mortality in urban and rural Tanzania. Lancet.

[14] Walker R, Whiting D, Unwin N (2010). Stroke incidence in rural and urban Tanzania: a prospective, community-based study. Lancet Neurol.

[15] World Health Organization (2013). A global brief on hypertension. Silent killer, global public health crisis.

[16] Seedat YK (2004). Recommendations for hypertension in sub-Saharan Africa. Cardiovasc J S Afr.

[17] Vorster HH (2002). The emergence of cardiovascular disease during urbanisation of Africans. Public Health Nutr.

[18] Kearney PM, Whelton M, Reynolds K (2005). Global burden of hypertension: analysis of worldwide data. Lancet.

[19] Ibrahim MM, Damasceno A (2012). Hypertension in developing countries. Lancet.

[20] Kerry SM, Micah FB, Plange-Rhule J (2005). Blood pressure and body mass index in lean rural and semi-urban subjects in West Africa. J Hypertens.

[21] Cappuccio FP, Kerry SM, Adeyemo A (2008). Body size and blood pressure: an analysis of Africans and the African diaspora. Epidemiology.

[22] Couser WG, Remuzzi G, Mendis S, Tonelli M (2011). The contribution of chronic kidney disease to the global burden of major non- communicable diseases. Kidney Int.

[23] Meisinger C, Doring A, Lowel H, KORA Study Group (2006). Chronic kidney disease and risk of incident myocardial infarction and all-cause and cardiovascular disease mortality in middle-aged men and women from the general population. Eur Heart J.

[24] Plange-Rhule J, Phillips R, Acheampong JW (1999). Hypertension and renal failure in Kumasi, Ghana. J Hum Hypertens.

[25] Cooper RS, Rotimi C, Kaufman JS (1998). Hypertension treatment and control in sub-Saharan Africa: the epidemiological basis for policy. Br Med J.

[26] Ibrahim MM, Rizk H, Appel LJ (1998). Hypertension prevalence, awareness, treatment, and control in Egypt. Results from the Egyptian National Hypertension Project (NHP). NHP Investigative Team. Hypertension.

[27] Pereira M, Lunet N, Azevedo A, Barros H (2009). Differences in prevalence, awareness, treatment and control of hypertension between developing and developed countries. J Hypertens.

[28] Cappuccio FP, Micah FB, Emmett L (2004). Prevalence, detection, management and control of hypertension in Ashanti, West Africa. Hypertension.

[29] Aburto NJ, Ziolkovska A, Hooper L (2013). Effect of lower sodium intake on health: systematic review and meta-analyses. Br Med J.

[30] Cappuccio FP, Capewell S (2015). Facts, issues and controversies in salt reduction for the prevention of cardiovascular disease. Funct Food Rev.

[31] Cappuccio FP, Capewell S, Lincoln P, McPherson K (2011). Policy options to reduce population salt intake. Br Med J.

[32] Kerry SM, Emmett L, Micah FB (2005). Rural and semi-urban differences in salt intake, and its dietary sources, in Ashanti, West Africa. Ethn Dis.

[33] Cappuccio FP, Plange-Rhule J, Phillips RO, Eastwood JB (2000). Prevention of hypertension and stroke in Africa. Lancet.

[34] Adeyemo AA, Prewitt TE, Luke A (2002). The feasibility of implementing a dietary sodium reduction intervention among free-living normotensive individuals in south west Nigeria. Ethn Dis.

[35] Cappuccio FP, Kerry SM, Micah FB (2006). A community programme to reduce salt intake and blood pressure in Ghana (IRSCTN 88789643). BMC Public Health.

[36] Cappuccio FP (1997). Ethnicity and cardiovascular risk: variations in people of African ancestry and South Asian origin. J Hum Hypertens.

[37] Brown MJ (2006). Hypertension and ethnic group. Br Med J.

[38] Modesti PA, Agostoni P, Agyemang C, European Society of Hypertension Working Group on Hypertension and Cardiovascular Risk Assessment in Subjects Living in or Emigrating from Low Resource Settings (2014). Cardiovascular risk assessment in low resources setting. A consensus document of the European Society of Hypertension Working Group on Hypertension and Cardiovascular Risk in Low Resource Settings. J Hypertens.

[39] Materson BJ, Reda DJ, Cushman WC (1993). Single-drug therapy for hypertension in men. A comparison of six antihypertensive agents with placebo. The Department of Veterans Affairs Cooperative Study Group on Antihypertensive Agents. N Engl J Med.

[40] ALLHAT Collaborative Research Group (2002). Major outcomes in high-risk hypertensive patients randomized to angiotensin-converting enzyme inhibitor or calcium channel blocker vs diuretic: the antihypertensive and lipid-lowering treatment to prevent heart attack trial (ALLHAT). JAMA.

[41] Buabeng KO, Matowe L, Plange-Rhule J (2004). Unaffordable drug prices: the major cause of non-compliance with hypertension medication in Ghana. J Pharm Pharm Sci.

[42] Harris TH, Twumasi-Abosi V, Plange-Rhule J, Cappuccio FP (2005). Hypertension management in Kumasi: barriers and prejudice?. J Hum Hypertens.

[43] World Health Organization (2005). Preventing chronic disease: a vital investment.

[44] Strong K, Mathers C, Leeder S, Beaglehole R (2005). Preventing chronic disease: how many lives can we save?. Lancet.

[45] Nugent R (2008). Chronic diseases in developing countries: health and economic burdens. Ann N Y Acad Sci.

[46] Alwan A, MacLean DR (2009). A review of non-communicable disease in low- and middle-income countries. Int Health.

[47] Lloyd-Williams F, O’Flaherty M, Mwatsama M (2008). Estimating the cardiovascular mortality burden attributable to the European Common Agricultural Policy on dietary saturated fats. Bull WHO.

